# Prenatal diagnosis of X‐linked myopathy associated with a *VMA21* gene mutation afforded through a novel targeted exome sequencing strategy applied in fetuses with abnormal ultrasound findings

**DOI:** 10.1002/ccr3.822

**Published:** 2017-02-04

**Authors:** Christopher Konialis, Efstratios Assimakopoulos, Birgitta Hagnefelt, Sophia Karapanou, Alexandros Sotiriadis, Constantinos Pangalos

**Affiliations:** ^1^ InterGenetics – Diagnostic Genetics CentreAthens11526Greece; ^2^ Genomis Ltd, Lynton HouseLondonWC1H 9BQUK; ^3^ 2nd Department of Obstetrics and GynecologyIppokrateion General HospitalAristotle University of ThessalonikiThessalonikiGreece

**Keywords:** Arthrogryposis, exome sequencing, fetal ultrasound, myopathy, prenatal diagnosis

## Abstract

Fetal malformations detected through routine prenatal ultrasound examination comprise a heterogeneous group potentially associated with genetic disorders where the underlying cause is difficult to establish. We present the prenatal diagnosis of a rare X‐linked myopathy involving a new VMA21 gene mutation, detected through a novel prenatal exome sequencing‐based approach.

## Introduction

Prenatal diagnosis of genetic disorders associated with structural fetal malformations and other fetal deformities is typically achieved through detailed ultasonographic (US) examination, while diagnosis of the precise nature of the disorder is often challenging due to considerable clinical and genetic heterogeneity 1. In these cases, array comparative genomic hybridization (aCGH), from a chorionic villus sample (CVS) or from amniotic fluid cells, is nowadays typically applied as a first‐tier test, with an average diagnostic yield of approximately 10–15% 2, 3. In a rather limited number of cases with characteristic ultrasound findings (e.g., very short long bones or increased nuchal translucency and normal karyotype), gene testing of one or few genes is applied, albeit with highly variable success rates. However, the underlying genetic cause of numerous other US anomalies, such as arthrogryposis, fetal akinesia, and genitourinary abnormalities, is rarely revealed through conventional genetic testing approaches commonly employed in a prenatal setting. This disappointing limitation has serious implications in terms of precise risk assessment in the course of an ongoing pregnancy, leading to a largely empirical and emotionally charged counseling of the parents. The aforementioned problem is exacerbated in pregnancies with recurrent fetal ultrasonographic abnormalities, where the failure to reach a definitive diagnosis in the previous pregnancy (often described as an isolated finding) is a common occurrence.

Whole exome sequencing (WES) approaches have very recently been described in malformed fetuses, mostly retrospectively in aborted fetuses products of conception (POC) and have provided encouraging results 4, 5, 6, 7, leading to discussions and debates as to the clinical usefulness of WES in the context of prenatal diagnosis 8.

Our group recently reported the initial findings of a novel expanded targeted exome sequencing testing strategy, designed for the investigation of fetuses with ultrasound abnormalities 9. This rapid prenatal testing approach – termed *Fetalis* – was applied in 14 fetal samples, involving three abortuses and 11 ongoing pregnancies, all with troubling ultasonographic findings, and a definitive or highly likely diagnosis was achieved overall in ~40% of cases 9. Follow‐up data of the eight apparently healthy newborns, born out of the ongoing pregnancy cases where *Fetalis* was performed as part of prenatal risk assessment, confirmed the negative results of the test.

In this report, we present the application of the *Fetalis* testing strategy in a prenatal case involving a fetus with arthrogryposis and micrognathia as the only recognizable ultrasound abnormalities, which led to the diagnosis of congenital X‐linked myopathy with excessive autophagy due to a novel *VMA21* gene mutation.

## Materials and Methods

A 25‐year‐old G2P1 pregnant woman in the 24th week of gestation presented for anomaly scan. In her previous pregnancy, 2 years before, multiple anomalies were detected in the fetus at 22 weeks, the main being micrognathia, short limbs, and arthrogryposis. Due to religious reasons, the parents had declined termination of pregnancy or further laboratory investigation, and the newborn male died shortly after birth. Both parents were healthy and nonconsanguineous, and the family history from both sides was unremarkable.

**Figure 1:**
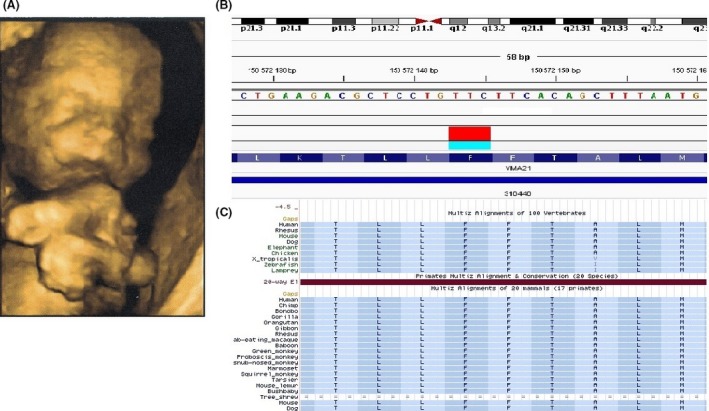
(A) Sample picture of level II ultrasound of male fetus. (B) IGV view of the *VMA21* gene c.94_96delTTC (p.Phe32del) nonframeshift deletion mutation. (C) UCSC Genome Browser alignments of the *VMA21* gene mutation region.

In the present second pregnancy, level II ultrasound examination revealed again the presence of micrognathia, short limbs, and arthrogryposis in the male fetus (Fig. 1A). The parents declined again termination of pregnancy, but due to reappearance of the same fetal abnormalities, they were counseled to have an amniocentesis for identifying a potential genetic abnormality through *Fetalis* testing. Following genetic counseling and informed consent, the family decided to proceed with genetic testing and an amniotic fluid sample was drawn and referred to our genetics clinic.

DNA was extracted from uncultured amniotic fluid cells, and *Fetalis* exome sequencing testing was performed as described previously 9. Briefly, genomic DNA was extracted from uncultured amniotic fluid cells, was subjected to whole exome DNA library construction with barcode incorporation, and massive parallel sequencing (MPS) was performed on the Ion Proton System utilizing a PI chip (Data S1). Annotated variants (Data S2) were subsequently imported for filtering, prioritization, and evaluation into a local Exome Management Application (*EMA*) custom pipeline (C Konialis & Z Agioutantis, manuscript in preparation) utilizing the *Fetalis* algorithm, targeting ~760 genes (Data S3) associated with genetic disorders which may present with fetal structural abnormalities 9.

Standard fluorescent bidirectional automated DNA Sanger sequencing was performed using the BigDye™ v3.1 Terminator Cycle Sequencing Kit (Applied Biosystems, Foster City, CA, USA) and analysis on the ABI PRISM™ 3730xl DNA sequencer (Applied Biosystems).

## Results

Analysis was completed in 8 days, and the results revealed the presence of a novel c.94_96delTTC (p.Phe32del) nonframeshift deletion mutation in the *VMA21* gene at Xq28 (Fig. 1B), occurring in a highly conserved region (Fig. 1C) in the transmembrane domain (amino acids 26–46) of this short 101 a.a. protein. The mutation is not present in the NHLBI Exome Variant Server (EVS), in ExAc and our local exome variant database (200 Greek exomes), while is predicted as deleterious by in silico tools (e.g., PROVEAN; Mutation Taster, www.mutationtaster.org/). Subsequent analysis by Sanger sequencing confirmed the hemizygous presence of the mutation in the male fetus and also revealed the heterozygous presence of the mutation in the mother, confirming X‐linked inheritance.

The *VMA21* gene encodes for an essential subunit of the vacuolar ATPase integral membrane protein and is required for the proper assembly and function of the V0 complex of the vacuolar ATPase proton pump in the endoplasmic reticulum. Mutations of the *VMA21* gene, leading to dysfunction of the proton pump, are associated with congenital X‐linked myopathy with excessive autophagy (OMIM 310440), presenting with severe muscle wasting, joint contractures, hypotonia, and respiratory insufficiency requiring ventilation 10. In a recent report 11, the authors describe their findings in a large kindred with seven affected males with congenital autophagic X‐liked myopathy, due to a splicing mutation in the *VMA21* gene, of which five died soon after birth due to inability to breathe and suckle 11. The clinical symptoms of marked hypoventilation are similar to those described for the previously affected male newborn in our family and taking also into consideration: (1) the presence of the *VMA21* gene mutation uncovered in the fetus and the carrier mother, and (2) the presence of arthrogryposis (joint contractures) in both offspring, a diagnosis of congenital X‐linked vacuolar myopathy was reached. The family were counseled regarding this finding in the fetus, and they decided not to terminate the ongoing pregnancy for religious reasons.

## Discussion

Although certain US abnormalities may be *suggestive* for the presence of a particular genetic disorder related with the observed malformations, the indications rarely lead to a definitive diagnosis in the course of pregnancy and prenatal diagnosis in future pregnancies centers once more on US examination. Recurrence of the abnormality(ies) in subsequent pregnancies provides a strong indication for an underlying genetic disorder, but this realization provides only empirical risk assessment and is of limited diagnostic value unless the precise molecular defect is recognized.

The presence of arthrogryposis in a fetus, typically detected through ultrasound examination in the 2nd or 3rd trimester of pregnancy, is a broad term used to describe multiple congenital contractures, which in turn are associated with numerous genetic disorders, such as myopathies, neuromuscular disorders, and metabolic disorders. Arthrogryposis may present as an isolated finding, or as part of a syndrome in combination with other congenital abnormalities. Similarly, myopathies and neuromuscular diseases constitute a diverse group of largely devastating disorders and are associated with significant infant and childhood mortality. Due to this considerable clinical and genetic heterogeneity, identification of the underlying genetic cause is a challenging task even in postnatal cases and is very rarely achieved in a prenatal setting. Furthermore, the recurrence risk is difficult to predict without precise knowledge of the genetic etiology, as genetic diseases presenting with fetal arthrogryposis may be expressed and inherited in all possible forms (recessive, dominant, X‐linked, mitochondrial).

Congenital X‐linked myopathy with excessive autophagy is an extremely rare disorder, with very few cases described up to date, mostly involving splicing mutations. In the case described herein, a prenatal diagnosis of this rare myopathy was afforded in a timely manner (10 days) through the *Fetalis* prenatal exome sequencing approach in an ongoing pregnancy, permitting accurate risk assessment and genetic counseling. To our knowledge, this is the first report of prenatal diagnosis of a myopathy “signaled” through an ultrasound finding in the course of pregnancy, through detection of a novel causative gene mutation not previously identified in affected or carrier family members. Furthermore, this report also highlights the clinical validity of the *Fetalis* exome sequencing strategy, designed specifically in order to expand our diagnostic capabilities in ongoing pregnancies with a malformed fetus.

## Conflict of Interest

No conflict of interests to report.

## Authorship

CK: conceived and designed the study, analyzed the data, and drafted the manuscript. EA: performed invasive procedures and clinical ultrasound evaluation, contributed in the collection and characterization of patient samples, and critical reading of the manuscript. BH and SK: performed DNA analysis, massive parallel sequencing procedures, and Sanger DNA sequencing. AS: contributed in clinical ultrasound evaluation, contributed in the collection and characterization of patient samples, and critical reading of the manuscript. CP: coordinated the study, performed clinical evaluation of the results, and contributed to the drafting of the manuscript.

## Supporting information


**Data S1.** Coverage analysis report.

**Data S2.** Annotated vcf file.

**Data S3.** Fetalis genes.
