# Supplementary material for: Prenatal diagnosis of X‐linked myopathy associated with a VMA21 gene mutation afforded through a novel targeted exome sequencing strategy applied in fetuses with abnormal ultrasound findings
Source: Clin Case Rep. 2017 Feb 4;5(3):308–11. doi: 10.1002/ccr3.822 (PMC5331204; doi:10.1002/ccr3.822)
Supplement: Supplementary file 1 — Data S1. Coverage analysis report. [file CCR3-5-308-s001.pdf]

Coverage Analysis Report

Reference Genome: hg19
Target Regions: AmpliSeqExome.20141113.designed

Number of mapped reads:	36,754,390
Percent reads on target:	75.53%
Number of amplicons:	293,903
Total assigned amplicon reads:	27,761,901
Percent assigned amplicon reads:	75.53%
Average reads per amplicon:	94.46
Uniformity of amplicon coverage:	92.12%
Amplicons with at least 1 read:	99.54%
Amplicons with at least 20 reads:	91.50%
Amplicons with at least 100 reads:	37.91%
Amplicons with at least 500 reads:	0.08%
Amplicons with no strand bias:	91.27%
Amplicons reading end-to-end:	37.19%
Total aligned base reads:	6,055,113,214
Total base reads on target:	4,878,469,311
Bases in target regions:	57,742,646
Percent base reads on target:	80.57%
Average base coverage depth:	84.49
Uniformity of base coverage:	90.99%
Target base coverage at 1x:	99.24%
Target base coverage at 20x:	88.83%
Target base coverage at 100x:	31.67%
Target base coverage at 500x:	0.06%
Target bases with no strand bias:	82.46%
Percent end-to-end reads:	60.83%
