# Supplementary material for: Prenatal diagnosis of X‐linked myopathy associated with a VMA21 gene mutation afforded through a novel targeted exome sequencing strategy applied in fetuses with abnormal ultrasound findings
Source: Clin Case Rep. 2017 Feb 4;5(3):308–11. doi: 10.1002/ccr3.822 (PMC5331204; doi:10.1002/ccr3.822)
Supplement: Supplementary file 3 — Data S3. Fetalis genes. [file CCR3-5-308-s003.pdf]

Fetalis genes

AAAS	BMP2	CXCR4	FBXL4	HOXA13	LHX4	OCRL	PRX	SHOC2	TMEM138
ABAT	BMP4	CYP11B1	FGD1	HOXA2	LIFR	OFD1	PSAP	SIX1	TMEM216
ABCC6	BMPER	CYP17A1	FGF10	HOXD13	LMBR1	OPHN1	PSAT1	SIX3	TMEM231
ABCD1	BMPR1B	CYP19A1	FGF17	HPGD	LMNA	ORC1	PTCH1	SIX5	TMEM237
ABCD3	BRAF	CYP21A2	FGF8	HRAS	LMNB1	ORC4	PTCH2	SIX6	TMEM5
ACAN	BRIP1	CYP2U1	FGF9	HS6ST1	LMX1B	ORC6	PTDSS1	SKI	TMEM67
ACO2	BSND	DARS	FGFR1	HSD17B3	LRAT	OTX2	PTEN	SLC12A1	TMEM70
ACTA1	BUB1	DCHS1	FGFR2	HSD17B4	LRP2	PAFAH1B1	PTH1R	SLC12A6	TNNI2
ACTB	BUB1B	DCX	FGFR3	HSPG2	LRP4	PALB2	PTPN11	SLC20A2	TNNT3
ACTG1	BUB3	DDHD2	FH	HYAL1	LZTFL1	PAX2	PVRL1	SLC25A19	TNXB
ADAMTS10	C12orf57	DDX59	FIG4	HYLS1	LZTR1	PAX3	PYCR1	SLC26A2	TP63
ADAMTS17	C5orf42	DHCR24	FKBP14	IBA57	MAP2K1	PAX6	RAB18	SLC2A10	TPM2
ADAMTSL2	CACNA1A	DHCR7	FKRP	ICK	MAP2K2	PCNT	RAB23	SLC35A2	TRAPPC9
ADAR	CASK	DHH	FKTN	IDS	MAP3K1	PDE4D	RAB3GAP1	SLC35A3	TREM2
ADGRG1	CBL	DHODH	FLNA	IDUA	MASP1	PDE6D	RAB3GAP2	SLC35D1	TREX1
ADSL	CC2D2A	DIS3L2	FLNB	IER3IP1	MBTPS2	PDGFB	RAB40AL	SLC6A8	TRIM32
AHI1	CCBE1	DKC1	FLRT3	IFIH1	MCOLN1	PDGFRB	RAD21	SLC9A6	TRIP11
AIMP1	CCDC28B	DLL3	FLT4	IFT172	MCPH1	PDHA1	RAD51C	SLX4	TRPV4
AIPL1	CCM2	DLX5	FLVCR2	IFT27	MECP2	PDHX	RAF1	SMAD3	TSC1
AIRE	CD96	DMD	FOXC1	IFT80	MED12	PDYN	RAI1	SMAD4	TSC2
AKR1C2	CDC6	DMPK	FOXC2	IFT88	MEF2C	PEX1	RAPSN	SMARCA4	TSEN34
AKT1	CDH1	DNM2	FOXE1	IGBP1	MEGF10	PEX10	RAX	SMARCB1	TSEN54
AKT3	CDKL5	DOCK6	FOXG1	IGHMBP2	MEOX1	PEX11B	RB1	SMARCE1	TSPYL1
ALDH1A3	CDKN1C	DOK7	FRAS1	IHH	MESP2	PEX12	RBM10	SMC1A	TTC8
ALMS1	CDON	DPYD	FREM1	IKBKG	MFRP	PEX13	RBM8A	SMC3	TUBA1A
ALPL	CDT1	DSP	FREM2	IL17RD	MGP	PEX14	RD3	SMOC1	TUBA8
ALX1	CEP164	DUSP6	FTO	IMPDH1	MID1	PEX16	RDH12	SMS	TUBB2B
ALX3	CEP290	DYM	FUZ	INPP5E	MIPOL1	PEX19	RECQL4	SNAP29	TUBB3
ALX4	CEP41	DYNC1H1	G6PC3	INSR	MKKS	PEX2	RELN	SNIP1	TUBGCP6
AMER1	CEP57	DYNC2H1	GAA	IQCB1	MKS1	PEX26	RET	SOS1	TULP1
AMT	CFTR	EARS2	GATA1	IRF6	MLH1	PEX3	RIPK4	SOX10	TWIST
ANKRD11	CHAT	EBP	GATA4	ISPD	MOCS1	PEX5	RIT1	SOX2	TWIST1
ANTXR2	CHD7	ECEL1	GATA6	ITGA6	MOCS2	PEX6	RMND1	SOX3	TYR
AP4B1	CHMP1A	EFNB1	GBA	ITGA8	MPL	PEX7	RMRP	SOX9	TYROBP
AR	CHN1	EFTUD2	GBA2	ITGB4	MPZ	PFKM	RNASEH2A	SPATA7	UBA1
ARFGEF2	CHRNA1	EGR2	GBE1	ITPR1	MRPS16	PGM1	RNASEH2B	SPECC1L	UBB
ARHGAP31	CHRNB1	EHMT1	GCSH	JAG1	MRPS22	PHF6	RNASEH2C	SPG11	UBE3A
ARID1A	CHRND	EIF2AK3	GDF1	JAM3	MSH2	PHF8	RNU4ATAC	SPRED1	UBE3B
ARID1B	CHRNE	EIF4A3	GDF3	JUP	MSH6	PIGA	ROR2	SPRY4	UFD1L
ARL13B	CHRNG	EMD	GDF5	KAL1	MSX1	PIGL	RPE65	SPTAN1	UPF3B
ARL6	CHST14	EMG1	GDF6	KANSL1	MSX2	PIGV	RPGRIP1	SRD5A2	UPK3A
ARVCF	CHST3	EMX2	GFAP	KAT6B	MTM1	PIK3CA	RPGRIP1L	SRD5A3	UTRN
ARX	CHSY1	EOGT	GFM1	KCNA1	MUSK	PIK3R2	RPL11	SRY	VANGL1
ASNS	CHUK	EP300	GJA1	KCNJ13	MVK	PIP5K1C	RPL15	STAC3	VANGL2
ASPA	CKAP2L	EPG5	GJB2	KCNJ2	MYBPC1	PITX1	RPL26	STAMBP	VAX1
ASPM	CNTN1	EPHX1	GJC2	KCNK9	MYH2	PKHD1	RPL35A	STAT3	VIPAS39
ASS1	COG4	ERBB3	GLDC	KCNQ1OT1	MYH3	PLCB4	RPL5	STS	VMA21
ASXL1	COL11A1	ERCC1	GLE1	KCNQ2	MYH8	PLEC	RPS10	STXBP1	VPS13B
ATL1	COL11A2	ERCC2	GLI2	KCNT1	NAA10	PLK4	RPS17	SUFU	VPS33B
ATM	COL18A1	ERCC4	GLI3	KCTD1	NBN	PLOD1	RPS19	SUMO1	VSX2
ATP6V0A2	COL1A1	ERCC5	GLUL	KCTD7	NDE1	PLOD3	RPS24	SYNE1	VTI1A
ATP7A	COL1A2	ERCC6	GMPPB	KDM6A	NEB	PLP1	RPS26	TACR3	WDPCP
ATP8A2	COL2A1	ERLIN2	GNAI3	KIAA0196	NEK1	PMM2	RPS6KA3	TAF2	WDR11
ATR	COL3A1	ESCO2	GNAO1	KIAA1279	NF1	PMP22	RPS7	TAZ	WDR19
ATRX	COL4A1	EVC	GNPTAB	KIAA2022	NFIX	PMS2	RTTN	TBC1D20	WDR34
ATXN10	COL5A1	EVC2	GNPTG	KIF14	NIN	PNKP	RUNX2	TBX1	WDR35
B3GALNT2	COL5A2	EYA1	GP1BB	KIF1A	NIPBL	POLR1C	RYR1	TBX15	WDR60
B3GALT6	COL6A1	EZH2	GPC3	KIF2A	NKX2-5	POLR1D	SACS	TBX22	WDR62
B3GALTL	COL6A2	FA2H	GPC6	KIF5C	NMNAT1	POLR3A	SALL1	TBX3	WDR81
B3GAT3	COL6A3	FAM111A	GPI	KIF7	NODAL	POMGNT1	SALL4	TBX4	WNT10B
B3GNT1	COL7A1	FAM123B	GPR56	KISS1R	NOG	POMGNT2	SAMHD1	TBX5	WNT3
B4GALT1	COL9A1	FAM20C	GPSM2	KMT2D	NOTCH2	POMK	SATB2	TBX6	WNT4
B9D1	COL9A2	FAM58A	GRHL3	KRAS	NPC1	POMT1	SC5D	TCF4	WNT5A
B9D2	COLEC11	FANCA	GRIP1	L1CAM	NPC2	POMT2	SCARF2	TCOF1	WNT7A
BBIP1	COMT	FANCB	GRM1	L2HGDH	NPHP1	POR	SDCCAG8	TCTN1	WT1
BBS1	COX7B	FANCC	GUCY2D	LAMA2	NPHP3	PORCN	SEC23A	TCTN2	WWOX
BBS10	CPT2	FANCD2	H19	LAMB1	NPR2	POU1F1	SEMA3A	TCTN3	ZBTB18
BBS12	CRB1	FANCE	HADHA	LAMC3	NR0B1	PQBP1	SEMA3E	TECPR2	ZDHHC9
BBS2	CREBBP	FANCF	HADHB	LARGE	NR5A1	PRDM5	SEPN1	TECT1	ZEB2
BBS4	CRH	FANCG	HCCS	LBN	NRAS	PRG4	SEPSECS	TFAP2A	ZFPM2
BBS5	CRLF1	FANCI	HDAC8	LBR	NSD1	PRKAR1A	SEPT9	TGFB3	ZFYVE26
BBS7	CRX	FANCL	HES7	LCA5	NSDHL	PROK2	SETBP1	TGFBR1	ZIC1
BBS9	CSPP1	FANCM	HESX1	LEMD3	NSMF	PROKR2	SF3B4	TGFBR2	ZIC2
BCOR	CTCF	FAT4	HIBCH	LFNG	NT5C2	PROP1	SH3PXD2B	TGIF	ZMPSTE24
BDNF	CTNS	FBN1	HIRA	LHB	OBSL1	PRRX1	SHANK3	TGIF1	ZNF423
BIN1	CUL7	FBN2	HOXA11	LHX3	OCLN	PRSS56	SHH	TMCO1	ZNF469
